# Intraspecific variation in response to magnitude and frequency of freeze-thaw cycles in a temperate grass

**DOI:** 10.1093/aobpla/plx068

**Published:** 2017-12-01

**Authors:** Charlotte C Dietrich, Juergen Kreyling, Anke Jentsch, Andrey V Malyshev

**Affiliations:** Biogeography, Bayreuth Center of Ecology and Environmental Research (BayCEER), University of Bayreuth, Bayreuth, Germany; Disturbance Ecology, Bayreuth Center of Ecology and Environmental Research (BayCEER), University of Bayreuth, Bayreuth, Germany; Experimental Plant Ecology, Ernst Moritz Arndt University Greifswald, Greifswald, Germany

**Keywords:** Above-ground net primary productivity (ANPP), central Europe, climate change, cocksfoot, *Dactylis glomerata*, freeze-thaw cycles, freezing-thawing, winter ecology

## Abstract

Winter warming and its accompanying predicted decrease in snow pack for northern temperate regions may increase frost damage to plants induced by an increase in freeze-thaw cycles (FTCs) due to reduced insulation. FTC frequency, minimum temperature during freezing and pre-existing local adaptations potentially all influence site-specific plant responses to future climatic changes. Within a chamber experiment, frost sensitivity towards recurrent FTCs was determined in 12 *Dactylis glomerata* populations from various European sampling sites differing in temperature and precipitation. After winter hardening, plants were frozen at −4 and −8 °C at frequencies of one, three and seven FTCs within a 1-week treatment phase. The control was kept at 4.5 °C. Plant survival, leaf elongation, chlorophyll content and above-ground net primary productivity (ANPP) decreased with lower minimum temperatures and higher FTC frequencies, while lower freezing temperatures generally proved more influential than increased freezing frequencies. Plant survival rates correlated with the amount of annual precipitation at seed origin, as individuals from comparably drier sites exhibited higher survival rates. This response, however, was limited in its effect to low freezing temperatures (−8 °C) and low and medium freezing frequencies (1 and 3 FTCs). In the set of surviving plants, water availability at seed origin best explained the plants’ growth responses to FTC treatment. The observed intraspecific variation emphasizes the ecological importance of potential local adaptations within a more variable future winter climate.

## Teaser

Promotional text: Freeze-thaw cycles (FTCs), which will become more common in vast regions where winter snow cover is decreasing due to climate change, negatively affect plant performance. In a FTC manipulation experiment with 12 ecotypes of a common temperate grass (Dactylis glomerata) from different European countries, freezing magnitude proved more detrimental than freezing frequency. Furthermore, individuals from drier origins exhibited higher survival rates. For plant injury, above-ground net primary productivity and chlorophyll content, treatment responses were similar across ecotypes. Local adaptations and within-species variation therefore might only provide a certain degree of buffering against adverse effects of (winter) climate change.

## Introduction

Climate change scenarios suggest an increase in the frequency of soil freeze-thaw cycles (FTCs) in many northern temperate regions, currently insulated and thermally stabilized by snow cover (Henry 2008; Pauli *et al.* 2013). As strong changes in seasonality and precipitation patterns accompany global warming, air frost (−30 to −45 %) and snow cover days (−30 to −40 %) will be strongly reduced across northern Europe (Jylhä *et al.* 2008). With ~50.5 % of the northern hemisphere’s total land area seasonally frozen (averaged for 1950–96; Zhang *et al.* 2003) a rapidly changing winter climate leaves low-stature ecosystems exposed to frost events, as the typically occurring and insulating snow cover decreases (Jylhä *et al.* 2008). Therefore, ecological processes related to snow cover and soil frost regimes are becoming increasingly important drivers of ecosystem functioning and composition within a warming and increasingly variable climate (Kreyling 2010; Pauli *et al.* 2013).

Freeze-thaw cycles can disrupt soil microorganism and soil aggregate dynamics, affecting below- and above-ground ecological processes (Oztas and Fayetorbay 2003; Schadt *et al.* 2003; Six *et al.* 2004; Sjursen *et al.* 2005; Vankoughnett and Henry 2013). Their effect on local plant populations however differs significantly depending on their timing, duration, severity and frequency. Short-term warming during winter may deharden acclimatized plant tissue within several days (Strimbeck *et al.* 1995; Kalberer *et al.* 2006) or even hours (Rapacz *et al.* 2000) increasing soil frost-related root injury upon (re-)freezing and in turn negatively affecting fine-root dynamics, primary productivity and nutrient cycling on-site (Fitzhugh *et al.* 2001; Tierney *et al*. 2001, 2003; Kreyling *et al.* 2008, 2010).

Within Europe’s temperate zone, perennial grass species are geographically widely distributed and grow under diverse climatic conditions (Beierkuhnlein *et al.* 2011). For several grass species, local adaptations to, e.g., precipitation patterns, climatic extremes and winter air temperatures have already been reported (Jentsch and Beierkuhnlein 2010; Kreyling *et al*. 2010, 2011; Malyshev *et al.* 2016), whereas local adaptations to FTCs have not yet been extensively studied. While winter seasonality typically evokes long-term physiological adaptations (Kreyling *et al*. 2010, 2011), short-term climatic events yield the potential to decimate entire populations (e.g. Jentsch *et al.* 2007). Thus, inherent local adaptation becomes a valuable strategy against increasing climatic variability (Kreyling *et al.* 2011).

Here, we tested for local preadaptation of 12 ecotypes to FTCs in cold-acclimated individuals of the temperate grass species *Dactylis glomerata*, spanning a pan-European climatic gradient within a climate chamber experiment. We hypothesized that (i) plant survival, chlorophyll content, leaf elongation and above-ground net primary productivity (ANPP) decrease with increasing magnitude and frequency of FTC manipulations, while plant injury increases. Furthermore, we also expected (ii) frost adaptation to vary among ecotypes with inherited local adaptation in frost tolerance being related to climatic conditions at seed origin, such as temperature, altitude and/or precipitation.

## Methods

### Plant material

**Figure 1.**
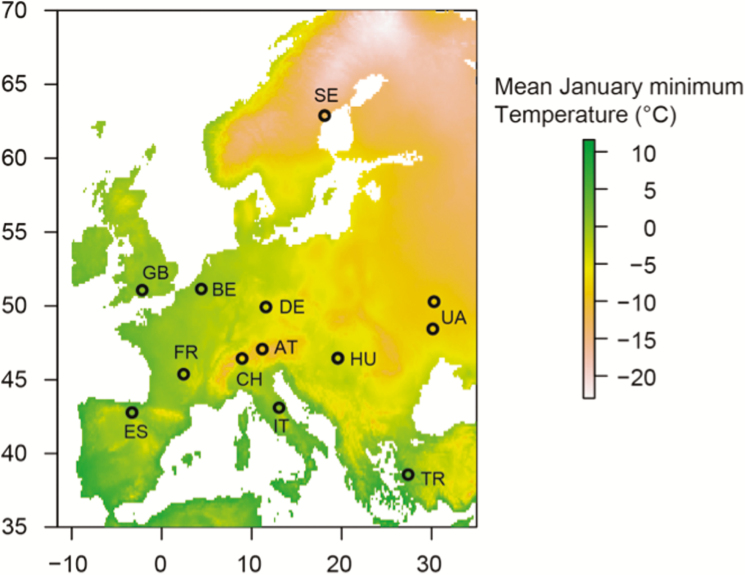
Map displaying the 12 seed origins of *Dactylis glomerata* used in the experiment. Colouring indicates the lowest temperature (°C) in January above European landmasses, as extracted from the WorldClim data set (Hijmans *et al.* 2005). Multiple sites of seed origin for single countries are displayed (see UA) but overlay for SE and ES.

In June 2013 (July for Swedish seeds), *D. glomerata* seeds were collected from European grasslands along a broad climatic gradient, where, to expert knowledge, the target species was autochthonous (Fig. 1). At least 20 individuals were sampled per site, with minimum sampling distances between sampled plants being >1 m. Corresponding climate data for each seed source were extracted from the WorldClim data sets (Hijmans *et al.* 2005; Table 1).

**Table 1.** Geographic and climatic characteristics at the 12 seed sampling sites, as extracted from WorldClim data sets (Hijmans *et al.* 2005). Shown are latitude (°, LAT, Northern direction), longitude (°, LON, Eastern direction), precipitation variability (PV), mean annual precipitation (mm, MAP), temperature of the warmest month (°C, TWM), temperature of the coldest month (°C, TCM), temperature of the warmest quarter (°C, TWQ), temperature of the coldest quarter (°C, TCQ), mean annual temperature (°C, MAT), altitude (m, ALT), temperature range (TR) and mean precipitation during the warmest quarter (mm, PWQ) and coldest quarter (mm, PCQ) for each site. Ukrainian and Spanish seeds originated from two, Swedish seeds from four locations with maximal distances between sampling sites of 200, 19.5 and 18 km, respectively.

	LAT	LON	PV	MAP	TWM	TCM	TWQ	TCQ	MAT	ALT	TR	PWQ	PCQ
Austria	47.07	11.18	28	1142	13.3	−10	8.3	−6.2	1.1	2041	7	406	146
Belgium	51.15	4.40	12	778	22.4	−0.2	17.1	3	10.2	9	7.7	198	75
Switzerland	46.44	8.94	25	1417	18.6	−5.3	13.3	−1.8	5.7	1290	6.9	468	163
Germany	49.92	11.58	20	674	22.5	−3.7	16.3	−0.3	8	426	8.4	221	76
Spain	42.77	−3.29	20	798	23.9	1	17.5	4.6	10.8	753	8.8	151	89
	42.76	−3.29	20	798	23.9	1	17.5	4.6	10.8	753	8.8	151	89
France	45.38	2.44	18	806	24.1	−1.4	16.9	2.8	9.9	596	10.3	218	94
Hungary	46.46	19.58	25	552	27.1	−8.3	20.2	0.5	10.8	123	9.6	176	72
Italy	43.10	13.05	18	881	26.6	0.5	20.5	4	12	649	7.5	194	101
Ukraine	48.44	30.13	31	590	24.7	−8	18.7	−3.8	7.7	195	8.2	214	83
	50.28	30.27	29	633	24.5	−8.4	18.8	−4.1	7.7	175	8.1	228	87
Sweden	62.94	17.79	24	656	20	−13	14.1	−7.9	3	105	8.5	191	74
	62.88	18.11	24	659	20.3	−12.8	14.4	−7.8	3.2	81	8.3	188	75
	62.87	18.11	24	659	20.3	−12.8	14.4	−7.8	3.2	81	8.3	188	75
	62.85	18.09	24	659	20.3	−12.8	14.4	−7.8	3.2	81	8.3	188	75
Turkey	38.56	27.39	73	769	31.6	1.1	26.3	5.8	14.4	503	11.4	35	157
UK	51.07	−2.16	19	804	20.8	0.3	15.3	3.5	9.1	145	7.5	169	90

Spanish and Ukrainian seeds originated from two locations, Swedish seeds from four different locations within their respective country of origin. The maximal distance separating these locations averaged 18 km for the Swedish, 19.5 km for the Spanish and 200 km for the Ukrainian seeds. The remaining nine ecotypes were represented by one location exclusively. Swedish, Spanish and Ukrainian climate parameters were averaged across their respective locations due to their close geographical proximity and/or comparative climate on site. The climatic data sets varied by a maximum of 0.4 °C mean annual temperature and 4 mm in annual precipitation for both the Swedish and Spanish locations. Ukrainian sampling sites exhibited a difference of 0.3 °C and 43 mm, respectively.

### Plant growth and cold acclimation

Individuals from all populations were grown under standardized conditions at the University of Bayreuth, Germany. Following germination in February 2014, plants were grown in seedling trays outside (protected from frost by glass frames) before being transplanted into pots measuring 5 cm in diameter and 7 cm in depth, filled with homogenized ED 73 substrate (Einheitserde Werkverband e.V.). Plants were watered at a rate that kept the soil constantly moist.

Six weeks after transplantation plants were cut to a height of 2 cm and placed into seven identical plant seed trays, later randomly partitioned to represent the control and the six treatment groups with five individuals per ecotype each (60 plants per tray). Tray positions were rearranged every other day. Plant arrangement within each tray was systematic, in order to control for edge and neighbouring effects between ecotypes. Plant cold acclimation commenced with a 10-h photoperiod, initiating cold acclimation in separate climate chambers (Adaptis by Conviron, model number: A1000) for all trays. The temperature was set to 6 °C during the night and 12 °C during the day. Plants were watered weekly and light intensity ranged between 70 and 120 µmol m^2^ s^1^. After 2 weeks, to enhance cold acclimation, the light duration was reduced to 8 h and the temperature to 4 °C during both day and night. Cold acclimation continued for 1 month, with 10 plants dying during this period.

### FTC manipulation

The FTC manipulations were administered in a three-factorial design, manipulating: (i) FTC frequency (zero for control plants and one, three and seven cycles each for the respective treatment trays), (ii) different minimum temperatures (4.5 °C for the control and −4 and −8 °C for each freezing treatment) and (iii) plant ecotypes (12 ecotypes). Each factorial combination was replicated five times, adding up to 420 plants in total.

**Figure 2.**
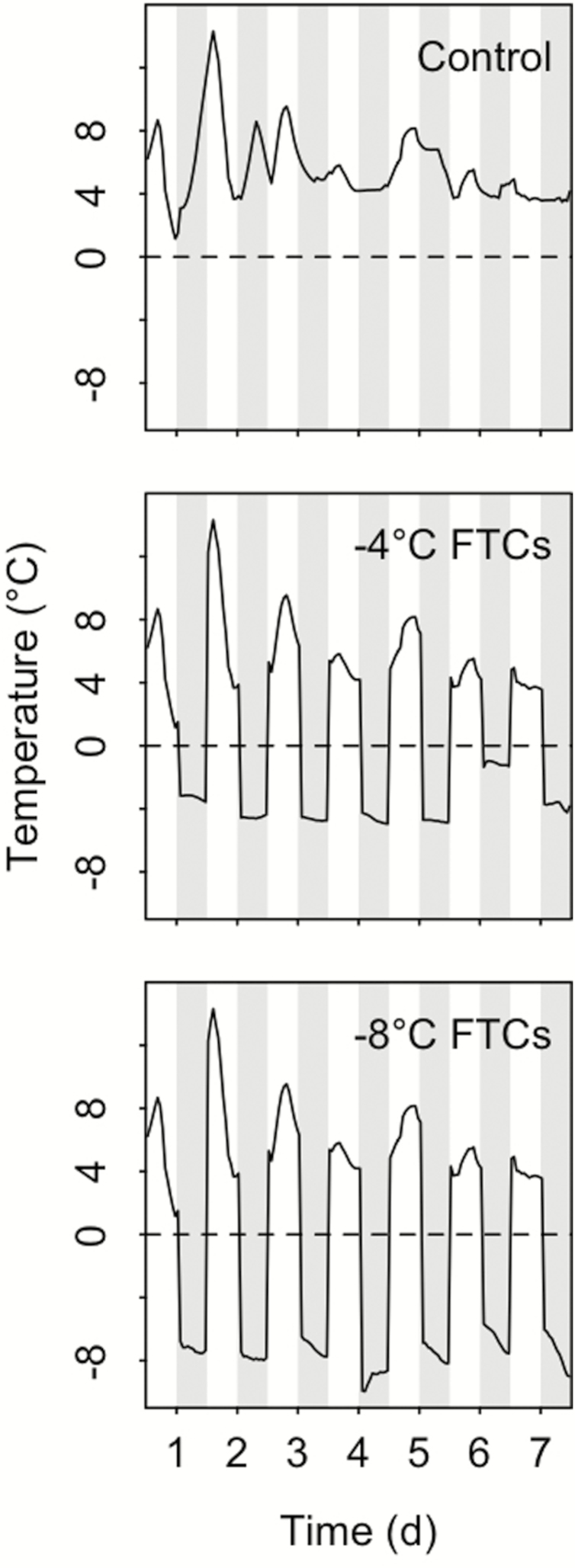
Running mean (*n* = 12) of the chamber temperatures measured hourly for control plants and plants experiencing freezing at −4 and −8 °C. Shown are all seven treatment days. Freezing periods are shaded grey.

Freeze-thaw cycle manipulations lasted a total of 7 days (Fig. 2). In order to simulate natural conditions, plants experienced thawing at 4.5 °C during the day and night-time freezing down to −4 or −8 °C in separate climate chambers (Fig. 2). For treatment, plants were moved from the control chamber (4.5 °C) to one of two treatment chambers set at either −4 or −8 °C. The temperature inside the chambers was measured using temperature loggers (HOBO Pro v2, Onset Computer Corporation, MA, USA). The last freezing cycle overlapped with the last day of treatment to affect all plants equally. Following the freezing treatments, plants were transferred into the greenhouse for a growth period of 2 weeks at an average mean air temperature of 20 °C and 43 % mean relative humidity during the day in order to simulate natural spring conditions.

### Response parameters

Initial plant survival was determined at the end of the 2-week post-frost growing phase in the greenhouse and transformed into relative survival rates for each factorial combination. Among the surviving plants, ANPP was quantified as the complete, dried harvest of above-ground biomass. In order to accurately estimate plant injury as a response to FTC manipulations, plant tissue was separated into categories of ‘living’ (green tissue) and ‘dead’ (brown tissue) based on visual inspection. It was subsequently dried for 48 h at 75 °C and weighed separately. Plant injury itself was later determined as percent dry mass of ‘dead’ tissue relative to overall ANPP.

Leaf lengths of predominantly green leaves were measured at the end of the treatment phase and at the end of the growth phase in the greenhouse. Both measurements were averaged for three different leaves of each plant. Leaf elongation was calculated by subtracting the mean leaf length at the end of the treatment phase from the average leaf length at the end of the greenhouse growing period to clearly distinguish treatment effects from pre-existing morphologies.

Leaf chlorophyll content was estimated using a SPAD 502 Plus Chlorophyll Meter (Konica Minolta). Measurements were repeated once at three different leaves at 1 (*t* = 1) and 2 weeks (*t* = 2) upon treatment completion and averaged, after we found no significant differences between the two sampling dates.

### Data analysis

In order to test for the effects of FTC frequency (freq), magnitude (magn) and seed origin (orig) on plant survival rates and within the set of surviving plants on ANPP, plant injury, leaf elongation and chlorophyll content three-factorial analyses of variance (ANOVAs) relating survival to freq, magn and orig were applied, with the latter being treated as a categorical factor. These analyses also tested for statistical interactions between all three factors and estimated their relative effect sizes (η_*p*_^2^; Cohen 1988) within the three-factorial ANOVA. Separate analyses to subsets of plants frozen at either −4 or −8 °C were applied in order to check for frequency or origin effects potentially masked in the three-factorial analyses.

To further identify the impact of inherited local adaptation on plant survival and plant parameter variation, the categorical factor of seed origin was transformed into numeric parameters of ecological importance, as fixed effects in interaction with freq and magn. Readily available factors used herein were: altitude, temperature of the coldest and warmest months, temperature of the coldest and warmest quarters, annual precipitation and its variability, mean annual temperature, annual temperature range (max. temperature of the warmest month − min. temperature of the coldest month) and the average precipitation values of the coldest and warmest quarters as extracted from the WorldClim data sets (Hijmans *et al.* 2005). The colinearity of these factors prohibited a direct comparison among them, but allowed for an independent identification of the variable(s) best explaining plant treatment responses.

In order to control for a possibly inflated type I error as a result of multiple comparisons, we applied false discovery rate testing (Storey *et al.* 2004). Subsequently, results with a *P*-value of α ≤ 0.05 and a corresponding *q*-value of α > 0.05 were disregarded as non-significant, as were results with a *P*-value of α > 0.05.

All response parameters were checked for their normal distribution using the Shapiro–Wilk test, visual inspection of frequency histograms and normal q-q plots. Both ANPP and leaf elongation values were consequently square-root transformed for the ANOVA analyses (non-transformed values are shown in the figures). All analyses were performed using R 3.4.3 (R Core Team 2017), installing additional ‘raster’ (Hijmans 2017), ‘heplots’ (Fox *et al.* 2017), ‘qvalue’ (Dabney and Storey 2017) and ‘rgdal’ packages (Bivand *et al.* 2017).

## Results

### Post-frost plant survival—FTC magnitude vs. frequency

The control plants exhibited the highest survival rate (96.7 %), followed by plants undergoing freezing at −4 °C (96.1 %) and −8 °C (62.5 %; Table 2). Survival rates among plants experiencing freezing at −4 °C decreased with increasing freezing frequency from 98.3 to 96.7 and 93.2 %. This effect was only partly observed in the −8 °C category: following the addition of two additional freezing cycles (one FTC vs. three FTCs) survival rates decreased by two-thirds, but did not further decrease upon the addition of four additional freezing cycles (three FTCs vs. seven FTCs).

**Table 2.** Absolute number of plant deaths following the treatment phase in response to both treatment type (FTC frequency and magnitude) and country of seed origin (*n* = 5 per factorial combination). Treatment combinations are sorted by temperature (4.5, −4 and −8 °C) and freezing frequency: low (one FTC), medium (med, three FTCs) and high (seven FTCs). The respective countries are ranked by the amount of annual precipitation received, ranging from lowest (Hungary) to highest (Switzerland), as extracted from the WorldClim data sets (Hijmans *et al.* 2005). The amount of annual precipitation on-site proved to be most influential on survival patterns and mortality following freezing treatment. In total, 72 out of 410 individuals died during treatment.

	4.5 °C	−4 °C			−8 °C			∑ = 72
	Control	Low	Med	High	Low	Med	High	
Hungary	1	1				2	1	5
Ukraine						2	1	3
Sweden						3	4	7
Germany					1	2	2	5
Turkey			1		1	3	3	8
Belgium					1	3	1	5
Spain						2	2	4
UK					2	1	2	5
France				2		1	4	7
Italy			1	1	1	2	2	7
Austria				1		3	2	6
Switzerland	1				3	3	3	10
∑ = 72	2	1	2	4	9	27	27	72

### Post-frost plant survival—climatic influence at seed origin

**Figure 3.**
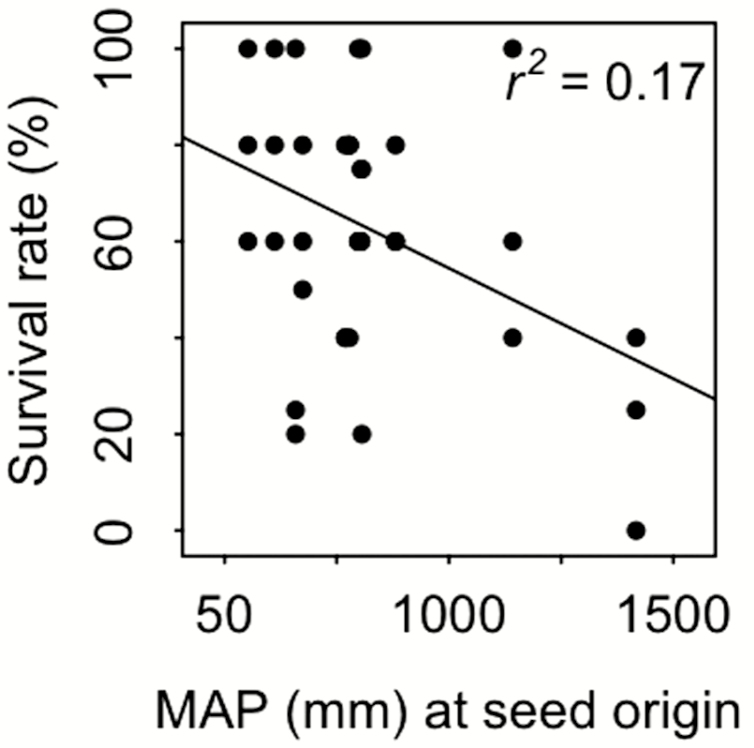
Survival rates for individuals frozen at −8 °C in dependence of mean annual precipitation values at the site of seed origin. Overall, plant survival decreases with an increase in mean annual precipitation values at the site of seed origin (*P* < 0.01, *r*^2^ ≈ 0.17, for individuals frozen at −8 °C independent of freezing frequency). Individuals adapted to a lower amount of annual precipitation at seed origin tended to be more resilient to freezing manipulations.

Mean annual precipitation at seed origin was the most influential climatic parameter explaining survival patterns across ecotypes (Fig. 3). Annual precipitation at the site of seed origin (*P* ≤ 0.01, η_*p*_^2^ = 0.08) and freezing magnitude (*P* ≤ 0.001, η_*p*_^2^ = 0.27) proved to have the strongest effect on survival rates. Freezing frequency × severity interactions (*P* ≤ 0.001, η_*p*_^2^ = 0.36) were significant. Typically, higher precipitation values at seed origin coincided with a decreased survival rate, as seen, for example, in the comparison of Hungary, exhibiting fairly low annual precipitation values with Switzerland representing the site with the highest amount of annual precipitation and the lowest survival rate overall (Table 2; Fig. 3). The effect of precipitation at seed origin on plant survival was most pronounced at low freezing temperatures (−8 °C) and low and medium freezing frequencies (three FTCs). Hence, ecotypes with a lower amount of annual precipitation at seed origin tended to be more resilient at lower freezing temperatures (see Table 2: Hungary, Ukraine, Sweden, Germany).

### Post-frost plant responses—FTC magnitude vs. frequency

Within the set of surviving plants (*n* = 338) all measured response parameters (ANPP, plant injury, leaf elongation, chlorophyll content) were significantly influenced by freezing magnitude and magnitude × frequency interactions (Table 3). In addition, chlorophyll content and plant injury were significantly affected by freezing frequency as a stand-alone factor.

**Table 3.** Statistical effects (*P*, *F*, η_*p*_^2^) of the three-factorial ANOVA for ANPP, plant injury, leaf elongation and chlorophyll content in dependence of the categorical factors seed origin (orig), freezing magnitude (magn) and frequency (freq) and any factorial combination thereof (orig × magn, orig × freq, magn × freq, orig × freq × mag). Bold face characters connote statistically significant responses. The significance level is set to *P* = 0.05.

	ANPP (g)			Plant injury (%)			Leaf elongation (cm)			Chlorophyll content		
	*P*	*F*	η_*p*_^2^	*P*	*F*	η_*p*_^2^	*P*	*F*	η_*p*_^2^	*P*	*F*	η_*p*_^2^
orig	0.166	1.4	0.05	0.196	1.4	0.06	**<0.001**	**7.3**	**0.26**	0.078	1.7	0.06
magn	**<0.001**	**73.8**	**0.15**	**<0.001**	**97.2**	**0.19**	**<0.001**	**57.7**	**0.16**	**<0.001**	**22.4**	**0.03**
freq	0.090	2.9	0.01	**<0.05**	**5.6**	**0.02**	0.126	2.4	0.01	**<0.01**	**9.0**	**0.03**
orig × magn	0.921	0.5	0.01	0.982	0.3	0.01	0.388	1.1	0.04	0.900	0.5	0.02
orig × freq	0.622	0.8	0.03	0.541	0.9	0.03	0.324	1.1	0.05	0.321	1.2	0.04
magn × freq	**<0.001**	**31.2**	**0.10**	**<0.001**	**49.6**	**0.14**	**<0.001**	**38.7**	**0.13**	**<0.001**	**27.5**	**0.09**
orig × freq × magn	0.424	1.0	0.04	0.778	0.7	0.02	0.459	1.0	0.04	0.813	0.6	0.02

**Figure 4.**
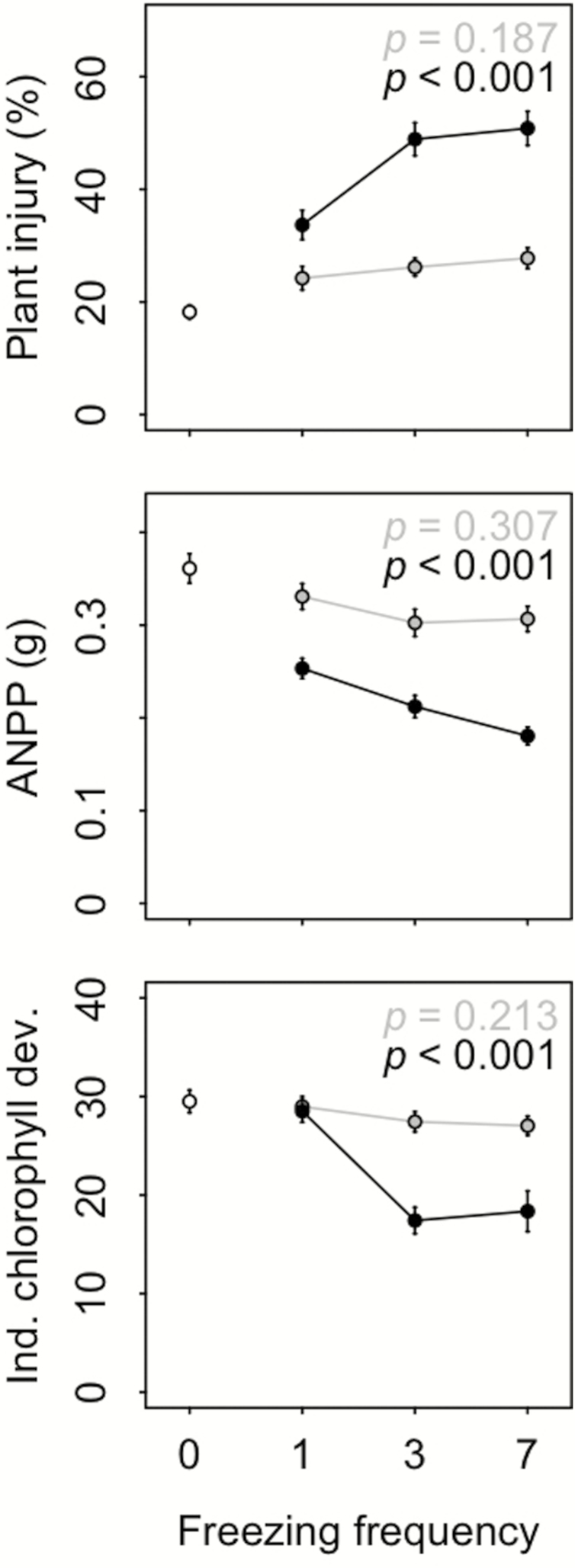
FTC manipulations affecting plant injury (%), above-ground primary productivity (ANPP; g) and indexed chlorophyll development in dependence of treatment temperature (4.5 °C = white, −4 °C = grey, −8 °C = black) and freezing frequency (*x*-axes). Displayed are mean values and their corresponding SEs over all ecotypes. *P*-values denote significance for parameter variation in dependence of freezing frequency for plants frozen at −4 °C (grey) and −8 °C (black).

Above-ground net primary productivity responded negatively to an increase in freezing magnitude, as it exhibited its lowest values at −8 °C. The effect of freezing frequency on ANPP resulted in a significantly increasing loss of primary productivity of 29.9, 41.2 and 50.0 % with increasing freezing frequency levels at low freezing temperatures (−8 °C; Fig. 4). Plant injury increased with increasing FTC magnitude and frequency. Compared to freezing frequency, the effect sizes of freezing magnitude proved to be higher for both ANPP (η_*p*_^2^ = 0.15 vs. η_*p*_^2^ = 0.01) and plant injury (η_*p*_^2^ = 0.19 vs. η_*p*_^2^ = 0.02).

Plants frozen at −8 °C exhibited significant leaf elongation losses of 50.5, 95.2 and 92.9 %, after one, three and seven FTCs, respectively. Increasing the FTC frequency from one to three cycles proved to have a more pronounced effect on leaf elongation variation than the further increase to seven cycles. The effect of freezing temperature (η_*p*_^2^ = 0.16) outweighed that of freezing frequency (η_*p*_^2^ = 0.01).

Mean chlorophyll content decreased with increasing freezing frequencies for both plants frozen at −4° and −8 °C in comparison to untreated individuals, with the latter exhibiting a lower chlorophyll content (Fig. 4). Chlorophyll content positively correlated with ANPP under freezing conditions. Here, freezing magnitude exhibited similar effect sizes (η_*p*_^2^ = 0.03) as freezing frequency (η_*p*_^2^ = 0.03).

### Post-frost plant responses—climatic influence at seed origin

**Figure 5.**
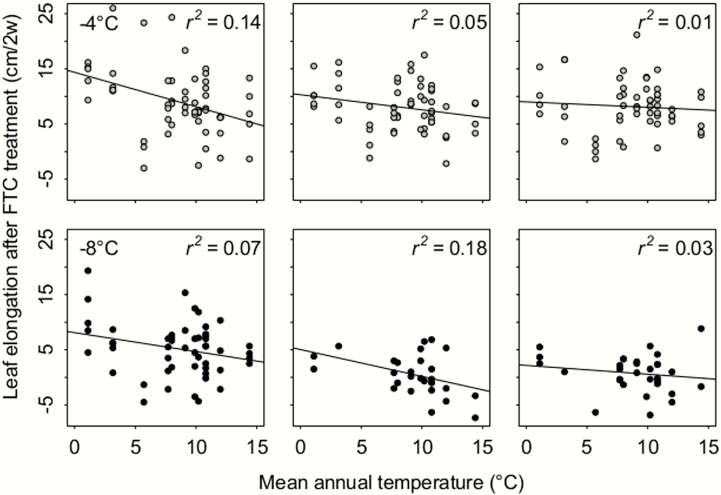
Leaf elongation data after 2 weeks of post-frost growth in the greenhouse for each of the six treatments (top row: −4 °C (grey), bottom row: −8 °C (black), from left to right: one, three and seven FTCs) in dependence of mean annual temperature values (°C) at the respective sites of seed origin. *R*^2^ values indicate the fit of the linear regression lines.

Following the FTC manipulations, ecotypes from comparatively colder climates grew faster than their counterparts (Fig. 5). Leaf elongation correlated negatively with the temperature of the warmest month, the warmest quarter and the annual mean temperature, as well as the temperature and precipitation of the coldest quarter at seed origin. Significant interactions between freezing treatment and climate conditions at seed origin were found for mean annual precipitation and precipitation of the warmest quarter with freezing frequency (Table 4).

**Table 4.** Overview over plant responses to treatment, including the control. The three-factorial ANOVA considers climatic conditions at the sites of seed origin, as well as freezing frequency and magnitude co-variates. Shown here are singular ecotype effects, as well as interactions between ecotype and either freezing magnitude (magn), freezing frequency (freq) or both (magn × freq). Effect directionality is denoted by +/−, with ‘+’ indicating a positive correlation between the environmental and plant parameters and ‘−’ a negative one. n.a. stands for ‘not available’ and denotes non-significant results. Environmental factors used herein are extracted from the WorldClim data sets (Hijmans *et al.* 2005) and include precipitation variability (PV), mean annual precipitation (mm, MAP), temperature of warmest month (°C, TWM), temperature of coldest month (°C, TCM), temperature of warmest quarter (°C, TWQ), temperature of coldest quarter (°C, TCQ), mean annual temperature (°C, MAT), altitude (m, ALT), temperature range (TR) and mean precipitation during the warmest quarter (mm, PWQ) and coldest quarter (mm, PCQ). Bold face characters indicate a statistically significant relationship. The significance level is set to *P* = 0.05 and *q* = 0.05.

	ANPP (g)				Plant injury (%)				Leaf elongation (cm)				Chlorophyll content			
	*P*	*F*	η_*p*_^2^	+/−	*P*	*F*	η_*p*_^2^	+/−	*P*	*F*	η_*p*_^2^	+/−	*P*	*F*	η_*p*_^2^	+/−
PV	0.549	0.4	0.00	n.a.	0.934	0.0	0.00	n.a.	0.250	1.3	0.01	n.a.	0.279	1.2	0.00	n.a.
PV × magn	0.519	0.4	0.00		0.448	0.6	0.00		0.443	0.6	0.00		0.771	0.1	0.00	
PV × freq	0.773	0.1	0.00		0.863	0.0	0.00		0.883	0.0	0.00		0.108	2.6	0.01	
PV × magn × freq	0.870	0.0	0.00		0.756	0.1	0.00		0.134	2.3	0.01		0.325	1.0	0.00	
MAP	0.320	0.9	0.00	n.a.	**<0.05**	**5.8**	**0.02**	**+**	0.056	3.7	0.01	n.a.	0.846	0.0	0.00	n.a.
MAP × magn	0.993	0.0	0.00		0.603	0.3	0.00		0.169	1.9	0.02		0.134	2.3	0.01	
MAP × freq	0.781	0.1	0.00		0.222	1.5	0.00		**<0.01**	**6.2**	**0.02**		0.819	0.1	0.00	
MAP × magn × freq	0.493	0.5	0.00		0.227	1.5	0.00		0.092	2.9	0.01		0.455	0.6	0.00	
TWM	0.736	0.1	0.00	n.a.	0.236	1.4	0.01	n.a.	**<0.01**	**10.3**	**0.04**	−	0.312	1.0	0.00	n.a.
TWM × magn	0.433	0.6	0.00		0.555	0.4	0.00		0.334	0.9	0.00		0.293	1.1	0.00	
TWM × freq	0.397	0.7	0.00		0.786	0.1	0.00		0.064	3.5	0.01		0.565	0.3	0.00	
TWM × magn × freq	0.969	0.0	0.00		0.264	1.3	0.00		0.671	0.2	0.00		0.929	0.0	0.00	
TCM	0.479	0.5	0.00	n.a.	0.215	1.5	0.01	n.a.	0.341	0.9	0.00	n.a.	0.853	0.0	0.00	n.a.
TCM × magn	0.449	0.6	0.00		0.833	0.0	0.00		0.062	3.5	0.01		0.413	0.7	0.00	
TCM × freq	0.957	0.0	0.00		0.263	1.3	0.00		0.838	0.0	0.00		0.514	0.4	0.00	
TCM × magn × freq	0.877	0.0	0.00		0.917	0.0	0.00		0.966	0.0	0.00		0.703	0.1	0.00	
TWQ	0.779	0.1	0.00	n.a.	0.295	1.1	0.00	n.a.	**<0.001**	**12.8**	**0.05**	−	0.252	1.3	0.00	n.a.
TWQ × magn	0.461	0.5	0.00		0.469	0.5	0.00		0.293	1.1	0.00		0.331	0.9	0.00	
TWQ × freq	0.341	0.9	0.00		0.811	0.1	0.00		0.072	3.3	0.01		0.391	0.7	0.00	
TWQ × magn × freq	0.960	0.0	0.00		0.194	1.7	0.01		0.662	0.2	0.00		0.896	0.0	0.00	
TCQ	0.352	0.9	0.00	n.a.	0.407	0.7	0.00	n.a.	**<0.01**	**10.4**	**0.04**	−	0.380	0.8	0.00	n.a.
TCQ × magn	0.511	0.4	0.00		0.747	0.1	0.00		0.123	2.4	0.00		0.752	0.1	0.00	
TCQ × freq	0.286	1.2	0.00		0.261	1.3	0.00		0.119	2.4	0.01		0.252	1.3	0.00	
TCQ × magn × freq	0.387	0.8	0.00		0.415	0.7	0.00		0.486	0.5	0.00		0.928	0.0	0.00	
MAT	0.485	0.5	0.00	n.a.	0.244	1.4	0.01	n.a.	**<0.001**	**13.9**	**0.05**	−	0.925	0.0	0.00	n.a.
MAT × magn	0.401	0.7	0.00		0.567	0.3	0.00		0.136	2.2	0.00		0.465	0.5	0.00	
MAT × freq	0.250	1.3	0.00		0.412	0.7	0.00		0.063	3.5	0.01		0.263	1.3	0.00	
MAT × magn × freq	0.625	0.2	0.00		0.226	1.5	0.00		0.563	0.3	0.00		0.985	0.0	0.00	
ALT	0.080	3.1	0.01	n.a.	0.235	1.4	0.01	n.a.	0.932	0.0	0.00	n.a.	0.707	0.1	0.00	n.a.
ALT × magn	0.770	0.1	0.00		0.395	0.7	0.00		0.240	1.4	0.01		0.147	2.1	0.01	
ALT × freq	0.855	0.0	0.00		0.470	0.5	0.00		0.143	2.2	0.01		0.729	0.1	0.00	
ALT × magn × freq	0.858	0.0	0.00		0.150	2.1	0.01		0.253	1.3	0.00		0.515	0.4	0.00	
TR	0.371	0.8	0.00	n.a.	0.052	3.8	0.01	n.a.	0.965	0.0	0.00	n.a.	0.968	0.0	0.00	n.a.
TR × magn	0.325	1.0	0.00		0.657	0.2	0.00		0.769	0.1	0.00		0.152	2.1	0.00	
TR × freq	0.947	0.0	0.00		0.711	0.1	0.00		0.074	3.2	0.01		0.178	1.8	0.01	
TR × magn × freq	0.771	0.1	0.00		0.938	0.0	0.00		0.622	0.2	0.00		0.671	0.2	0.00	
PWQ	0.275	1.2	0.00	n.a.	0.107	2.6	0.01	n.a.	0.787	0.1	0.00	n.a.	0.846	0.0	0.00	n.a.
PWQ × magn	0.784	0.1	0.00		0.781	0.1	0.00		0.555	0.4	0.01		0.169	1.9	0.01	
PWQ × freq	0.745	0.1	0.00		0.533	0.4	0.00		**<0.05**	**6.4**	**0.02**		0.895	0.0	0.00	
PWQ × magn × freq	0.940	0.0	0.00		0.251	1.3	0.00		0.646	0.2	0.00		0.964	0.0	0.00	
PCQ	0.960	0.0	0.00	n.a.	0.377	0.8	0.00	n.a.	**<0.05**	**5.4**	**0.02**	−	0.956	0.0	0.00	n.a.
PCQ × magn	0.638	0.2	0.00		0.416	0.7	0.00		0.228	1.5	0.00		0.837	0.0	0.00	
PCQ × freq	0.858	0.0	0.00		0.598	0.3	0.00		0.963	0.0	0.00		0.343	0.9	0.00	
PCQ × magn × freq	0.436	0.6	0.00		0.721	0.1	0.00		<0.05	4.1	0.01		0.226	1.5	0.01	

Plant injury positively correlated with the amount of mean annual precipitation at seed origin (*P* ≤ 0.05, η_*p*_^2^ = 0.02). Ecotypes experiencing a comparatively low amount of annual precipitation tended to be less sensitive to injury at −4 °C.

## Discussion

Survival of *D. glomerata* individuals across populations of different geographic origin was negatively affected by lower freezing temperatures and increasing freezing frequencies, most likely due to frost-induced tissue damage and dieback (Bokhorst *et al.* 2009; Wipf *et al.* 2009). Notably, increasing freezing frequencies from three to seven FTCs did not further decrease survival chances for plants frozen at −8 °C, which suggests that freezing frequency is limited in its effect, with additional FTCs not being more detrimental following three FTCs. This supposed limitation of freezing frequency was mirrored in subsequent response analyses of chlorophyll content, leaf elongation, plant injury and ANPP among the surviving individuals, as freezing magnitude outweighed freezing frequency in its respective effect size.

Overall, survival rates, primary production, leaf elongation and chlorophyll content decreased with freezing magnitude, while plant injury increased. This increase of frost damage at temperatures likely to occur within a snow-free environment stresses the importance of diminishing soil insulation. Without snow, the lack of insulation leaves above- and below-ground biota exposed to freezing temperatures (Fitzhugh *et al.* 2001; Hardy *et al.* 2001; Maljanen *et al.* 2007). This may result in an increase of fine-root necromass and an overall decrease of root biomass within the organic layer (Groffman *et al.* 2001; Gaul *et al.* 2008). A plant’s ability to take up nutrients is in turn reduced (Campbell *et al.* 2014). In agreement with our findings, recent field experiments in temperate grasslands indicate that this loss of insulation and an exposure to cyclic freezing result in a decrease in ANPP for at least one growing season (Schuerings *et al.* 2014).

Out of the 11 climatic parameters used to characterize the locations of seed origin, the amount of annual precipitation correlated best with a population’s survival chances. Plants from comparably drier sites exhibited higher survival rates. While this effect was prominent at −8 °C and low and medium freezing frequencies, independent treatment effects increased at high freezing frequencies. This indicates that, while certain plants are better equipped to survive a short amount of time (here 12 h) under frost stress, conditions of recurrent freezing at −8 °C exceed ecotype-specific tolerances. Previous experiments with shrubs (Boorse *et al.* 1998) and woody plants (Nardini *et al.* 2000; Nilsson 2001; Rehfeldt *et al.* 2002) have revealed patterns of intraspecific variation in the cold sensitivity of plants corresponding to the environmental conditions of their origin. In addition, Poirier *et al.* (2012) showed that following a growing season under conditions of restricted water supply, temperate *D. glomerata* populations exhibited lower amounts of induced frost damage the following winter than Mediterranean populations. Physiological plant responses to drought and frost stress are similar at cellular levels (Beck *et al.* 2007), implying cross-stress tolerance (Walter *et al.* 2013), i.e. drought-adapted plants being more frost-tolerant. Detecting species and ecotypes which show superior cross-stress tolerance and stress memory might therefore be a potential adaptation strategy in agriculture against the projected increase in climate-driven extreme weather events (IPCC 2012).

Furthermore, our results imply that while temperature might be an influential factor in determining overall plant growth patterns, a populations’ reaction to freezing is much more likely to be dependent on precipitation patterns at seed origin, as interactions between freezing manipulations and all precipitation-related parameters (MAP, PWQ, PCQ) proved significant. Northern ecotypes grew faster following freezing, likely due to a higher frost tolerance accumulated prior to treatment. Recently, Malyshev *et al.* (2014) have shown that following exposure to colder temperatures southern grass ecotypes cold acclimate at a slower rate, maintaining growth longer than their northern counterparts. This potentially beneficial growth advantage however increases the plants’ vulnerability, as subsequent frost events threaten to injure lesser cold-acclimated tissue. This is of special importance, as FTCs are likely to increase especially in regions where the current mean winter temperature is close to the freezing point (Henry 2008).

## Conclusions

Herein we show that FTCs, which will become more common in vast regions where winter snow cover is decreasing, negatively affect plant performance. Freezing magnitude proved more detrimental than freezing frequency. Our results also suggest that a populations’ survival chances are likely to be dependent on precipitation patterns at seed origin, as individuals from drier origins exhibited higher survival rates. Among surviving plants, leaf elongation patterns were linked to water availability year-round, as well as during the warmest and the coldest quarters. For plant injury, ANPP and chlorophyll content, treatment responses were similar across ecotypes. Local adaptations and within-species variation therefore might only provide a certain degree of buffering against adverse effects of (winter) climate change.

## Sources of Funding

This work is part of the project SIGNAL which is funded by the ERA-Net BiodivERsA (http://www.biodiversa.org; funding ID 01LC1201).

## Contributions by the Authors

A.J. and J.K. conceived the project; A.V.M., J.K. and C.C.D. designed and set up the manipulation experiment; C.C.D. sampled and analysed the data; C.C.D. wrote the paper with substantial input from A.V.M. and contributions from all authors.

## Conflict of Interest

None declared.
